# A comparative study of CTG monitoring one hour before labor in infants born with and without asphyxia

**DOI:** 10.1186/s12884-023-06040-3

**Published:** 2023-10-26

**Authors:** Seyedeh Tala Nabipour Hosseini, Fatemeh Abbasalizadeh, Shamsi Abbasalizadeh, Sanaz Mousavi, Paria Amiri

**Affiliations:** 1 Women’s Reproductive Health Research Center, Department of Perinatology, Faculty of Medicine, Tabriz University of Medical Sciences, Tabriz, Iran; 2 School of Nursing and Midwifery, Tabriz University of Medical Science, Tabriz, Iran

**Keywords:** Fetal cardiotocogram, Uterine contraction, Asphyxia

## Abstract

**Background and Aim:**

Asphyxia is a condition arising when the infant is deprived of oxygen, causing Fetal brain damage or death, which is associated with hypoxia and hypercapnia. Although fetal Cardiotocography (CTG) can show the Fetal health status during labor, some studies have reported cases of fetal asphyxia despite reassuring CTGs. This study hence aimed to compare FHR Monitoring and uterine contractions in the last hour before delivered between two groups of infants born with and without asphyxia.

**Methodology:**

The study was conducted on 70 pregnant women who delivered Taleghani and Al-Zahra academic teaching hospitals of Tabriz for labor in 2020–2021.

**Results:**

The study data showed no significant difference between mothers of infants with and without asphyxia in terms of demographics (p > 0.05). The prevalence of asphyxia was significantly higher only in mothers with the gravidity of 3 and 4 (p = 0.003). In terms of the methods for labor induction, the use of oxytocin was more common among mothers of infants with asphyxia (74.3%) than in those of infants without asphyxia (p = 0.015). The results also revealed a significant difference between infants with and without asphyxia in the Apgar score (first, fifth, and tenth minutes), need for neonatal resuscitation, umbilical cord artery Acidosis (pH, bicarbonate, and BE), and severity of HIE between two groups of infants with asphyxia and without asphyxia (p < 0.0001). The comparison of fetal CTG 0 to 20 min before the delivery indicated that normal variability was observed in 71.4% of infants born with asphyxia, whereas this figure for infants born without asphyxia was 91.4% (p = 0.031). However, the results showed no significant difference between the two groups of infants in any of the tstudied indicators at 20 and 40 min before the labor(p > 0.05). There was a significant difference between the two groups of infants in terms of deceleration at 40 and 60 min before the labor, as it was observed in 53.6% of infants born with asphyxia and only 11.1% of those born without asphyxia. The results also demonstrated a significant difference between the two groups in the type of deceleration (p = 0.025). Pearson and Spearman correlation coefficients showed a significant and direct relationship between interpretation the CTG of the three Perinatologists(p < 0.0001, r > 0.8).

**Conclusion:**

The study results demonstrated a significant difference between infants born with asphyxia and those born without asphyxia in variability at 0 to 20 min before the labor and deceleration at 40 to 60 min before the labor.

## Introduction

Asphyxia is a condition arising when the infant is deprived of oxygen, causing fetal brain damage or death, which is associated with hypoxia and hypercapnia. Depending on the severity of hypoxia, this condition can cause ischemia and destructive effects on vital fetal organs such as the heart, lungs, kidneys, and, most importantly, the brain [1]. Placental abruption, uterine rupture, umbilical cord prolapse, obstructed labor, improper use of oxytocin, and fetal infections are among the conditions that can lead to intrauterine hypoxia-ischemia [2]. Despite advances in perinatal, obstetric, and neonatal care, childbirth injuries are still the second most common cause of infant mortality (24%) after prematurity [3].

About 4 million infants annually die due to asphyxia around the world, which accounts for 23% of all infant mortalities and 8% of all cases of death among children [1]. The prevalence of asphyxia varies from country to country, as it is equal to one in every 1000 infants in developed countries but 5–10 in every 1000 infants in developing countries [4]. In a study conducted in Bojnurd, Iran, the prevalence of asphyxia was shown to be 2.1% [5]. Such data further highlight the necessity of preventing and controlling this medical problem.

As described earlier, the main cause of asphyxia is the insufficient supply of oxygen to the fetus [1], which can be the result of severe uterine contractions. It has been reported that severe uterine contractions are responsible for 75% of cases of asphyxia during labor [6]. According to most international Obsterical associations, the upper limit of normal uterine contractions is defined as five contractions in ten minutes (each lasting for 30 s, on average) [7]. Nowadays, topography devices make it possible to record uterine contractions from the surface of the abdomen [8]. Therefore, they are commonly used to evaluate uterine contractions during labor and delivery [9].

recording fetal heart rate (FHR) through Electronic fetal monitoring is the easiest and firth method of modern fetal health assessment in obstetrical [10]. During delivery In this test, fetal heart activity and uterine contractions are recorded simultaneously and continuously to predict fetal health [11]. A 10-minute CTG is considered reassuring (Category 1) when it shows a specific pattern including the baseline (110–160), at least two acceleration lines (an increase of 15 beats for 15 s) due to fetal movements, and beat-to-beat variability in the range of 6–25 beats [12]. It is generally assumed that the reduced variability is the most valid sign of fetal distress [13]. On the other hand, FHR responses to uterine contractions may indicate uterine perfusion or placental function [14].

Periodic changes in FHR can be classified as follows: early deceleration (a slowing of the fetal heart rate starting at the beginning of the contraction, and returning to the baseline by the end of the contraction), late deceleration (a visually apparent, gradual decrease in the fetal heart rate typically following the uterine contraction), variable deceleration (it is not related to uterine contractions and occurs in response to umbilical cord obstruction during labor), long-term deceleration (it lasts more than two minutes and less than 10 min), and sinusoidal pattern (smooth, sine-like pattern visible with periodic repetitions of 3 to 5 beats per minute seen on the ECG for 20 min or more) [13].

A CTG is considered Non reassuring (Category 3) when it contains at least one of the following items: absent variability along with late decelerations, absent variability along with variable decelerations, absent variability along with bradycardia for at least ten minutes, and sinusoidal pattern for at least 20 min. Such conditions can predict possible fetal asphyxia [12, 15]. These patterns have a dynamic state during labor, as they can quickly alternate between reassuring and non reassuring pattern [12]. Therefore, clinical fetal monitoring during labor aims to ensure that enough oxygen is supplied to the fetus in a way that it can endure the delivery process. Although the widespread use of fetal monitoring has failed to reduce the prevalence of cerebral palsy, there is enough evidence that it can reduce fetal and neonatal mortalities [16]. A study on fetal ECGs during the last hour before delivery showed that even fetuses with a reassuring CTG (Category 1) may develop fetal acidosis [17]. This study aims to compare cardiogram in the last hour before delivery between two groups of infants born with and without asphyxia (Based on AAP, ACOG guidelines).

## Methodology

The statistical population in this descriptive-analytical study consisted of all women admitted to Taleghani and Al-Zahra teaching hospitals of Tabriz for delivery in 2020–2021. The participants were selected from among the women aged 18–45 years who were admitted to the maternity ward of the studied centers with labor pains or indications for induction of labor and delivered to live newborns (with or without fetal asphyxia). According to a study by Thomas et al. (2011) [18] and assuming α = 0.05, p-value = 0.5, and error coefficient = 12%, the sample size was calculated to be 70. After selecting the participants, they were assigned to two groups of 35: mothers of newborns with asphyxia and mothers of newborns without asphyxia.

### Sampling

The participants of this study were selected from Taleghani and Al-Zahra teaching hospital of Tabriz, which are affiliated with Tabriz University of Medical Sciences. The sampling process began after obtaining permission from the Ethics in Research Committee of Tabriz University of Medical Sciences (code IR.TBZMED.REC.1400.260). The sampling process began in May 2020 and finished in March 2021. The research team selected the participants based on the inclusion and exclusion criteria and monitored them after obtaining their permission and informed consent. All participants in this study were continuously monitored by medical staff. Cardiotocography (CTG) information, including baseline FHR, baseline FHR variability, and possible FHR accelerations or decelerations, over the last hour before the delivery, and demographic information of mothers and newborns health status were recorded on special a checklist.

### Study population

The inclusion criteria were being the onset of labor pains, reaching the due date or medical indication of termination of pregnancy, not taking drugs that affect the heart of the fetus such as magnesium sulfate, narcotics and painkillers before delivery, not being addicted to drugs and smoking and alcohol. The exclusion criteria were addiction to drugs and smoking and alcohol consumption, taking drugs that affect the heart of the fetus such as magnesium sulfate, narcotics, painkillers and Fever and any factor that causes Tachycardia before delivery.

### Data collection tools

The required data were collected using a two-part questionnaire. The first part consisted of items about demographics and obstetrical information of participants as well as the maternity information and health status of newborns, which was completed based on the medical records of participants. The second part of the questionnaire was designed for the evaluation of FHR and uterine contractions, which was completed in three phases: 0 to 20minuts before delivery, 20 to 40 min before delivery, and 40 to 60 min before delivery. The CTG checklist was developed based on the system proposed by the National Institute of Child Health and Human Development (NICHD) in 1997 for the classification of FHR patterns [12]. This checklist was completed by three experienced perinatologists of the Department of Obstetrics and Gynecology, Tabriz University of Medical Sciences, who were unaware of the participant assignment.

### Statistical analysis

The obtained data were statistically analyzed in SPSS-22. Since the data followed an abnormal pattern of distribution, they were reported as frequency (%), mean (± standard deviation), and median. The normal distribution of data was examined using the Kolmogorov - Smirnov test and, if necessary, the Shapiro–Wilk test. The relationship between qualitative variables was tested using chi-square and, if necessary, Fisher’s exact test. Other methods of inferential statistics, including the student’s t-test and the Mann–Whitney U test, were also employed for data analysis [19–21]. The significance level was determined to be p < 0.05.

## Results

This study was conducted on two groups of 35: mothers of newborns with asphyxia and mothers of newborns without asphyxia. There was no significant difference between the two groups in demographic variables, such as maternal age, gestational age, parity, maternal weight, maternal height, and maternal BMI (p > 0.05). However, a significant difference was observed between the two groups in gravidity, as the prevalence of asphyxia was significantly higher among newborns of mothers with the gravidity of 3 and 4 (p = 0.003) (Table 1).

**Table 1:** Summary of the demographic status of the studied mothers

Variable		group		P
		No asphyxiation	asphyxiation	
age		27.69 (6.38)	29.11 (6.22)	0.346 ǂ
Gestational age^¥^		38.42 (38–40)	39.14(40-38.14)	0.106^£^
gravity	1	27 (77.1)	22 (62.9)	0.003^*^
	2	2 (5.7)	12 (34.3)	
	3	3 (8.6)	1 (2.9)	
	4	3 (8.6)	0 (0.0)	
parity	0	27 (77.1)	22 (62.9)	0.59^*^
	1	4 (11.4)	12 (34.3)	
	2	3 (8.6)	1 (2.9)	
	3	1 (2.9)	0 (0.0)	
mother’s weight^¥^		80 (75–88)	85 (78–88)	0.266^£^
mother’s height^¥^		168 (165–170)	168 (165–170)	0.434^£^
BMI^¥^		29.4 (27.5–31.2)	30.1 (27.9–30.9)	0.593^£^

* Fisher’s exact test ǂ Student’s t-test £ Mann-Whitney test

¥ Median (interquartile range) € mean (Standard deviation)

The results also showed no significant difference between the two groups for types of labor induction (p > 0.05). However, the use of oxytocin was more common among mothers of newborns with asphyxia (74.3%) than in those of newborns without asphyxia (45.7%) (p = 0.015) (Table 2).

**Table 2:** The status of indicators related to childbirth in the studied mothers

Variable		group		P
		No asphyxiation N = 35	Asphyxiation N = 35	
The cause of childbirth	The onset of labor pains	15 (42.9)	14 (40.0)	0.375^*^
	Delivery due date	6 (17.1)	6 (17.1)	
	Arrival of due date + termination of indicated pregnancy	3 (8.6)	6 (17.1)	
	dripping	10 (28.6)	7 (20.0)	
	Preeclampsia	1 (2.9)	0 (0.0)	
	Vaginal bleeding	0 (0.0)	2 (5.7)	
Induction to terminate pregnancy	Yes	28 (80.0)	23 (65.7)	0.179^£^
	No	7 (20.0)	12 (34.3)	
Method used	intrauterine catheter	3 (8.6)	4 (11.4)	1.000^*^
	Misoprostol	1 (2.9)	5 (14.3)	0.198^*^
	Oxytocin	26 (74.3)	16 (45.7)	0.015^£^
Presence of concurrent disease	Yes	12 (34.3)	10 (28.6)	0.797^£^
	No	23 (65.7)	25 (71.4)	
Type of disease	Insulin-dependent diabetes	0 (0.0)	1 (2.9)	1.000^*^
	Non-insulin dependent diabetes	2 (5.7)	4 (11.4)	0.673^*^
	Chronic hypertension	0 (0.0)	2 (5.7)	0.493^*^
	Gestational hypertension	5 (14.3)	5 (14.3)	1.000^*^
	preeclampsia	4 (11.4)	0 (0.0)	0.114^*^
Growth retardation	Yes	1 (2.9)	1 (2.9)	1.000^*^
	No	0 (0.0)	0 (0.0)	
Repeat caesarean section	Yes	0 (0.0)	1 (2.9)	1.000^*^
	No	35 (100.0)	34 (97.1)	
Type of delivery	NVD	7 (20.0)	8 (22.9)	0.771^£^
	C.S	28 (80.0)	27 (77.1)	
Use of vacuum	Yes	0 (0.0)	2 (5.7)	0.493^*^
	No	35 (100.0)	33 (94.3)	
Cause of caesarean section	Irregularity of the fetal heart	20 (71.4)	21 (77.8)	0.823^*^
	lack of progress	4 (14.3)	4 (14.8)	
	Fetal heart irregularity + lack of progress	4 (14.3)	2 (7.4)	

* Fisher’s exact test £ Chi-square test

The results examining the health status of newborns indicated no significant difference between newborns with and without asphyxia in multi-organ system failure (MOSF), birth weight, and zero Apgar score (p > 0.05). Nevertheless, a significant difference was found between the two groups in Apgar score at the first, fifth, and tenth minutes, resuscitation operations, blood cord analysis at delivery (pH, bicarbonate, and BE), and severity of HIE (p < 0.0001) (Table 3).

**Table 3:** Status of the variables related to the studied infants

Variable		group		P
		No asphyxiation N = 35	Asphyxiation N = 35	
Newborn Apgar^¥^	first minute	7 (6–8)	2 (1–3)	< 0.0001^£^
	The fifth minute	8 (8–10)	4 (3–5)	< 0.0001^£^
	10th minute	10 (10–10)	5 (5–7)	< 0.0001^£^
Death of a baby (Apgar zero)	first minute	0 (0.0)	0 (0.0)	
	The fifth minute	0 (0.0)	2 (5.7)	0.493^*^
	10th minute	0 (0.0)	3 (8.6)	0.239^*^
Resuscitation in the delivery room	Yes	15 (42.9)	35 (100.0)	< 0.0001^*^
	No	20 (57.1)	0 (0.0)	
Umbilical cord ABG result at birth^¥^	PH	7.3 (7.3–7.27)	6.8 (6.9–6.5)	< 0.0001^£^
	Bicarbonate	19 (19–20)	15 (12.9–17)	< 0.0001^£^
	BE	-8 (-7 _ -9)	-18 (-15 _ -20)	< 0.0001^£^
Multi organ failure, newborn	Yes	1 (2.9)	6 (17.1)	0.106^*^
	No	34 (97.1)	29 (82.9)	
Severity of HIE	does not have	35 (100.0)	7 (20.0)	< 0.0001^*^
	mild	0 (0.0)	21 (60.0)	
	medium	0 (0.0)	4 (11.4)	
	intense	0 (0.0)	3 (8.6)	
Baby’s weight€		3278.9 (410.7)	3167.6 (396.6)	0.253 ǂ

* Fisher’s exact test ǂ Student’s t-test £ Mann-Whitney test ¥ Median (interquartile range) € mean (Standard deviation)

About agreement between the three physicians who evaluated the baseline FHR at 0–20, 20–40, and 40–60 min before the delivery, Pearson and Spearman correlation coefficients reveal a significant and direct relationship between evaluations conducted by the three physicians (Table 3). Correlation coefficients also suggested a high level of agreement between the three physicians (p < 0.0001, r = 0.8). When it comes to the agreement between the three physicians who evaluated the CTG status (variability, acceleration, deceleration, type of deceleration, and category) at 40–60 and 0–20 min before the delivery, the Cramér’s V coefficients showed a significant and direct relationship between evaluations conducted by the three physicians. This means that there was a high level of agreement between the three physicians (p < 0.0001). However, no significant relationship was observed between the evaluations of the three physicians at 20–40 min before the delivery (p > 0.05) (Table 4).

**Table 4:** Measurement of the agreement between the evaluations of three doctors assessing the Baseline status one last hour before delivery in the studied infants

Variable	Measurement time	Assessing physician *	Pearson’s correlation coefficient^£^	P - value
BASELINE	40 to 60 min before delivery	Doctor 1 - Doctor 2	0.848	< 0.0001
		Doctor 2 - Doctor 3	0.862	< 0.0001
		Doctor 1 - Doctor 3	0.869	< 0.0001
	20 to 40 min before delivery	Doctor 1 - Doctor 2	0.941	< 0.0001
		Doctor 2 - Doctor 3	0.842	< 0.0001
		Doctor 1 - Doctor 3	0.945	< 0.0001
	0 to 20 min before delivery	Doctor 1 - Doctor 2	0.875 ǂ	< 0.0001
		Doctor 2 - Doctor 3	0.840 ǂ	< 0.0001
		Doctor 1 - Doctor 3	0.845 ǂ	< 0.0001
Variable			Kramer’s coefficient v	P - value
VARIABILITY	40 to 60 min before delivery		0.274	< 0.0001
	20 to 40 min before delivery		0.041	0.867
	0 to 20 min before delivery		0.230	< 0.0001
ACCEIERATION	40 to 60 min before delivery		0.421	< 0.0001
	20 to 40 min before delivery		0.121	0.285
	0 to 20 min before delivery		0.430	< 0.0001
DECELERATION	40 to 60 min before delivery		0.561	< 0.0001
	20 to 40 min before delivery		0.005	0.998
	0 to 20 min before delivery		0.409	< 0.0001
TYPE OF DECELERATION	40 to 60 min before delivery		0.440	< 0.0001
	20 to 40 min before delivery		0.197	0.207
	0 to 20 min before delivery		0.406	< 0.0001
CATEGORY	40 to 60 min before delivery		0.407	< 0.0001
	20 to 40 min before delivery		0.052	0.923
	0 to 20 min before delivery		0.330	< 0.0001

* Doctor 1: Shamsi Abbas Alizadeh Doctor 2: Fatemeh Abbas Alizadeh Doctor 3: Sanaz Mousavi

£ Pearson’s correlation coefficient ǂ Spearman’s correlation coefficient

The evaluation of fetal CTGs in terms of baseline FHR, acceleration, deceleration, and type of deceleration at 0 to 20 min before the labor showed no significant difference between newborns with asphyxia and those without asphyxia (p > 0.05). However, there was a significant difference between the two groups of newborns in FHR variability, as normal variability was observed in 71.4% of newborns born with asphyxia and in 91.4% of those born without asphyxia (p = 0.031). The evaluation of fetal CTGs at 20 to 40 min before the labor also indicated no significant difference between the two groups of newborns in any of the above-mentioned variables (p > 0.05). The study results on the evaluation of fetal CTGs at 40–60 min also demonstrated that there was no significant difference between newborns with and without asphyxia in baseline FHR, variability, and acceleration (p > 0.05). However, a significant difference was observed between the two groups in terms of deceleration, as it was observed in 53.6% of newborns born with asphyxia and only 11.1% of those born without asphyxia. There was also a significant difference between the two groups of newborns in the type of deceleration (p = 0.025) (Table 5).

**Table 5:** Comparison of fetal ECG status in the group of babies born with asphyxia and without asphyxia

Variable			group		P
			No asphyxiation N = 18	Asphyxiation N = 28	
BASELINE			142.84 (8.35)	142.39 (19.97)	0.917£
40 to 60 min before delivery	VARIABILITY	NORMAL	17 (100.0)	27 (96.4)	1.000*
		ABSENT	0 (0.0)	1 (3.6)	
	ACCEIERATION	YES	13 (72.2)	18 (64.3)	0.575 ǂ
		NO	5 (27.8)	10 (35.7)	
	DECELERATION	YES	2 (11.1)	15 (53.6)	0.004 ǂ
		NO	16 (88.9)	13 (46.4)	
	TYPE OF DECELERATION	Early Deceleration	16 (88.9)	13 (52.0)	0.025*
		Variable deceleration	0 (0.0)	1 (4.0)	
		Prolong Deceleration	2 (11.1)	10 (40.0)	
	CATEGORY	І	0 (0.0)	1 (4.0)	0.008 ǂ
		II	13 (72.2)	10 (40.0)	
		III	5 (27.8)	1 (4.0)	
Variable			N = 18	N = 28	P
BASELINE			135.77 ± 6.75	133.94 ± 29.94	0.747£
20 to 40 min before delivery	VARIABILITY	NORMAL	100(28)	27(90)	0.238*
		ABSENT	0(0)	3(10)	
	ACCEIERATION	YES	22(78.6)	18(60)	0.127 ǂ
		NO	6(21.6)	12(40)	
	DECELERATION	YES	14(50)	18(60)	0.444 ǂ
		NO	14(50)	12(40)	
	TYPE OF DECELERATION	NO	14(53.3)	12(44.1)	0.816*
		Early Deceleration	4(15.4)	5(17.2)	
		Variable deceleration	8(30.8)	11(37.9)	
		Prolong Deceleration	0(0)	1(3.4)	
	CATEGORY	І	11(40.7)	9(31)	0.579 ǂ
		II	16(59.3)	19(65.5)	
		III	0(0)	1(3.4)	
Variable			N = 28	N = 30	p
BASELINE			(128.3–140) 131.7	(106.7–140) 130	0.371^£^
20 to 40 min before delivery	VARIABILITY	NORMAL	32(91.4)	25(71.4)	0.031^£^
		ABSENT	3(8.6)	10(28.6)	
	ACCEIERATION	YES	8(22.9)	9(25.7)	0.78^£^
		NO	27(77.1)	26(74.4)	
	DECELERATION	YES	33(94.3)	33(94.3)	1^£^
		NO	2(5.7)	2(5.7)	
	TYPE OF DECELERATION	NO	2 (6.1)	2(7.4)	0.363*
		Early Deceleration	0(0)	3(11.1)	
		Variable deceleration	6(18.2)	5(18.5)	
		Prolong Deceleration	17(51.5)	12(44.4)	
	CATEGORY	І	8(24.2)	4(14.8)	0.028*
		II	0(0)	1(3.7)	
		III	2(5.7)	1(2.9)	

## Discussion and conclusion

The study results showed that the prevalence of asphyxia was significantly higher among newborns of mothers with the gravidity of 3 and 4. Based on the literature review, there was no similar study to confirm this finding. However, this result can be justified by the fact that the increased gravidity is usually associated with aging, conditions such as high blood pressure, and other pregnancy complications.

The study results indicated that there was no significant difference between mothers of newborns with asphyxia and those of newborns without asphyxia in some variables such as the for labor induction, indications for termination of pregnancy, the method used (catheter and misoprostol), comorbidities, type of comorbidities, history of previous cesarean section, type of delivery, instrumental for for delivered, and the indication for cesarean section. There was a significant difference between them only in terms of the use of oxytocin, as the use of oxytocin was more common among mothers of newborns with asphyxia than in those of newborns without asphyxia.

In a study conducted by Abubakri et al. (2019), it was shown that there was a significant relationship between only two demographic variables, i.e. anemia and improper meals, and fetal asphyxia [22]. Jiang et al. (2019) also reported no significant relationship between asphyxia and some demographic variables such as age, gender, and birth weight [23]. Samad et al. (2016) also showed that the prevalence of asphyxia was higher among the newborns of mothers who had received inadequate prenatal care and those who had a home birth or dystocia [24]. These results are consistent with the findings of this study regarding the relationship between demographics and asphyxia.

The study results demonstrated no significant difference between newborns born with and without asphyxia in terms of baseline FHR, accelerations, decelerations, and type of decelerations from 0 to 20 min before the labor. However, there was a significant difference between them in FHR variability, as normal variability was observed in 71.4% of newborns born with asphyxia and in 91.4% of those born without asphyxia. At 20 to 40 min before the labor, no significant difference was observed between newborns born with and without asphyxia in any of the studied variables. The results also revealed a significant difference between the two groups of newborns in FHR decelerations at 40 to 60 min before the labor, as they were observed in 53.6% of newborns born with asphyxia and only 11.1% of those born without asphyxia. There was also a significant difference between them in the type of decelerations.

Warmerdam et al. (2016) compared 14 fetuses with asphyxia and 14 healthy fetuses in order to study the effects of FHR variability during labor on fetal distress. They reported that there was no significant difference between the two groups of fetuses with and without asphyxia in beat-to-beat (short-term) variability if the periods of uterine contractions and rest are not differentiated. However, a significant difference was observed between them in this regard when uterine contractions were differentiated from periods of rest [25]. Because the number of uterine contractions increases as the time of labor approaches, it can be stated that this result is consistent with the findings of this study regarding the beat-to-beat variability. Abbasalizadeh et al. (2015) investigated the relationship between Non reassuring patterns of fetal cardiotocography and asphyxia and reported that there was no significant difference between fetuses with asphyxia and those without asphyxia in FHR accelerations, but there was a significant difference between them in terms of FHR variability. In other words, minimum or no FHR variability was observed in most fetuses with asphyxia. They also showed that late decelerations were significantly more common among fetuses with asphyxia, despite their normal variability [26]. Leszczynska-Gorzelak et al. (2002) also reported the same results [27]. As mentioned earlier, it is generally assumed that the reduced variability of baseline FHR variability is the most valid sign of fetal distress [13]. Although the literature review indicated that a few studies have meticulously examined fetal health status in three periods of the last hour before the labor, the results of the above-mentioned studies corroborated the findings of this study.

One of the strengths of this study was the meticulous evaluation of fetal CTGs in three periods of 20 min during the last hour before the labor, something that was rarely done in previous studies. Another strength of this study was the evaluation of fetal CTGs by three different specialists. Observance of all principles of research and blinding was among other strengths of this study. The only weakness of this study was the absence of the corresponding author (the main researcher) during the labor of some participants, and, as a result, CTG were performed by midwives, interns, or residents.

## Conclusion

The study findings demonstrated a significant difference between newborns born with asphyxia and those born without asphyxia in FHR variability at 0 to 20 min before the labor and FHR deceleration at 40 to 60 min before the labor.

## Data Availability

Datasets used and analyzed during this study are available from the corresponding author on reasonable request.
